# Supplementary material for: Stacking Model‐Based Classifiers for Dealing With Multiple Sets of Noisy Labels
Source: Biom J. 2025 Mar 12;67(2):e70042. doi: 10.1002/bimj.70042 (PMC11898607; doi:10.1002/bimj.70042)
Supplement: Supplementary file 1 — Supporting Information [file BIMJ-67-e70042-s001.zip › density-based-ensemble-model-main/competitors_real_data/netlab-master/netlab-master/help/fevbayes.htm]

Netlab Reference Manual fevbayes


# fevbayes

## Purpose

Evaluate Bayesian regularisation for network forward propagation.

## Synopsis

```
extra = fevbayes(net, y, a, x, t, x_test)
[extra, invhess] = fevbayes(net, y, a, x, t, x_test, invhess)
```

## Description

`extra = fevbayes(net, y, a, x, t, x_test)` takes a network data structure
`net` together with a set of hidden unit activations `a` from
test inputs `x_test`, training data inputs `x` and `t` and
outputs a matrix of extra information `extra` that consists of
error bars (variance)
for a regression problem or moderated outputs for a classification problem.
The optional argument (and return value)
`invhess` is the inverse of the network Hessian
computed on the training data inputs and targets. Passing it in avoids
recomputing it, which can be a significant saving for large training sets.

This is called by network-specific functions such as `mlpevfwd` which
are needed since the return values (predictions and hidden unit activations)
for different network types are in different orders (for good reasons).

## See Also

`mlpevfwd`, `rbfevfwd`, `glmevfwd`

---

**Pages:**
Index


---

Copyright (c) Ian T Nabney (1996-9)
